# Insulin inhibits melanoma tumor growth through the expression of activating transcription factor 4, without detectable expression of transcription factor CHOP: an *in vivo* model^⋆^

**DOI:** 10.1016/j.abd.2023.07.012

**Published:** 2024-04-23

**Authors:** Daniel do Prado, Marianna Boia-Ferreia, Hanna Camara da Justa, Andrea Senff-Ribeiro, Sérgio Lunardon Padilha

**Affiliations:** a Department of Internal Medicine, Universidade Federal do Paraná, Curitiba, PR, Brazil; b Department of Cell Biology, Universidade Federal do Paraná, Curitiba, PR, Brazil

Dear Editor,

In the first half of the 20^th^ century, there were reports^1^ of size reduction in different types of tumors treated with high doses of insulin, including a case of metastatic melanoma.^2^ As these treatments induced hypoglycemic states^2^ and due to the close relationship between neoplasms and glycemia,^3^ it was concluded that this response occurred due to patients low blood glucose levels. However, the mechanism of this interaction seems to be the expression of activating transcription factor 4 (ATF4) and other associated proteins, such as transcription factor CHOP (TFCHOP).

Therefore, the present study aims to demonstrate the effects of insulin administered to mice with melanoma, the relationship between the tumor response and blood glycemia and the protein expression possibly involved in this mechanism.

All procedures were approved by the Animal Use Ethics Committee (number 23075.067738/2019-05). Murine melanoma cell lines (B16-F10) were obtained from ATCC (American Type Culture Collection, Manassas, VA, USA), cultured in DMEM medium, and injected subcutaneously (6 × 10^6^ cells per animal) into male C57BL/6 mice. aged eight to 12 weeks (obtained from Instituto Carlos Chagas, Fiocruz – Paraná), totaling 50 animals. The mice were fed a standard diet (Purina) during the experiments. After five days, the development of a solid tumor of variable size was observed at the site of application. Regular human insulin 100 IU/mL (Novolin R®) was used to treat the animals, diluted in 50% glucose solution. The mice were divided into four groups according to the treatment scheme: 15 mice in the control group (0.1 mL of 50% glucose solution); ten mice in the 1IU/kg group (0.1 mL of solution at 0.1 IU/mL); ten mice in the 2IU/kg group (0.1 mL of solution at 0.2 IU/kg); and 15 mice in the 4IU/kg group (0.1 solution at 0.4 IU/mL). Insulin was administered intraperitoneally for a total of 15 days, divided into three cycles of five days, with two-day intervals without hormone administration (Supplementary Material 1). Glycemic levels were measured using a glucose meter (Accu Check Active, Roche Diagnostic, Germany), using a blood sample obtained through a small cut in the tail. The collection occurred only in the control, 2IU, and 4IU groups, three times a week. After the end of treatment, the animals were euthanized using a combination of xylazine hydrochloride and ketamine hydrochloride (10%) in 50 mcL (1:1).

Tumor tissue was extracted and the immunohistochemical analysis was performed using slides of tumor sections from B16-F10 tumors obtained from *in vivo* experiments. Immunohistochemistry slides were prepared according to the indexed protocol (Supplementary Material 2) using the primary antibodies: anti-TFCHOP for rats (Santa Cruz Biotechnology, cat.sc 7351) and anti-ATF4 for rats, (Santa Cruz Biotechnology, cat. sc 390063). Immunohistochemical quantification was performed using the “ImageJ Analysis Software” program, according to the Crowe and Yue protocol.^4^ Control slides were used to determine basic parameters and as a reference for relative expression. The immunohistochemistry positivity area and the number of nuclei per evaluated slide field were also assessed. Three ×20 magnification fields per slide were used for the analysis.

The statistical analysis and graphs were carried out using the R program (R Development Core Team). A comparison was performed using the Kruskal-Wallis test and revised using Dunn’s test for tumor sizes and protein expression, nuclei count and stained area on the slides. Glycemic levels were not included in the statistical analysis, since the aim of the study was not to demonstrate differences between the groups regarding this variable, but rather the absence of severe hypoglycemia.

The effects of insulin *in vivo* were analyzed using the animal model, measuring the size of the tumors at the end of the treatment. The box-plot graph of the size of the tumors is shown in Fig. 1A, while the glycemic levels obtained in the animals are shown in Fig. 1B, considering the reference value for mice (150 mg/dL).

**Figure 1:**
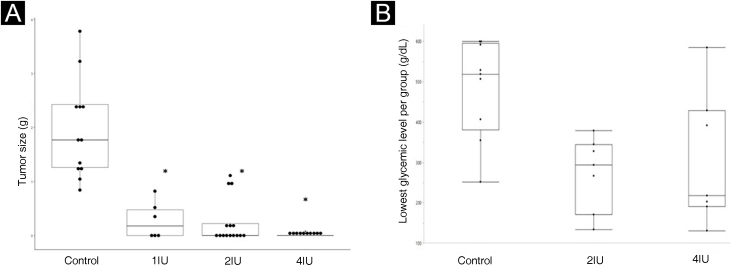
Insulin inhibits tumor growth in a murine model of melanoma (C57BL6/B16F10), without causing hypoglycemia. (A) Boxplot representing the average size of tumors excised in each experimental group. Statistical evaluation was performed using the Kruskal-Wallis test, followed by Dunn’s test. (B) Boxplot representation of glycemic levels measured in mice. The 1IU group did not have blood glucose levels assessed due to the fact that it was a standard dose used in medical practice. The lowest blood glucose levels, in any of the groups, were not below 136 mg/dL, showing the absence of hypoglycemia in the mice. * p value < 0.05 in relation to the control group.

The number of animals that died during the experiments comprised two in the control group and four in the 1IU/kg group. Deaths were due to tumor progression in the animals. These deaths were observed throughout the second week of the experiments and the tumors of these animals were not analyzed.

The expression of ATF4 (Fig. 2A and B) was increased in the 2IU/kg and 4IU/kg groups when compared to the control group, with statistical significance. The images obtained to evaluate TFCHOP (Fig. 2C and D) showed a statistical difference in the 4IU group when compared to the control group (Fig. 3). In the qualitative analysis of the slides, a lower count of nuclei was observed in the treated groups and a larger stained area in the 4IU (ATF4) and 1IU (TFCHOP) groups (Table 1).

**Figure 2:**
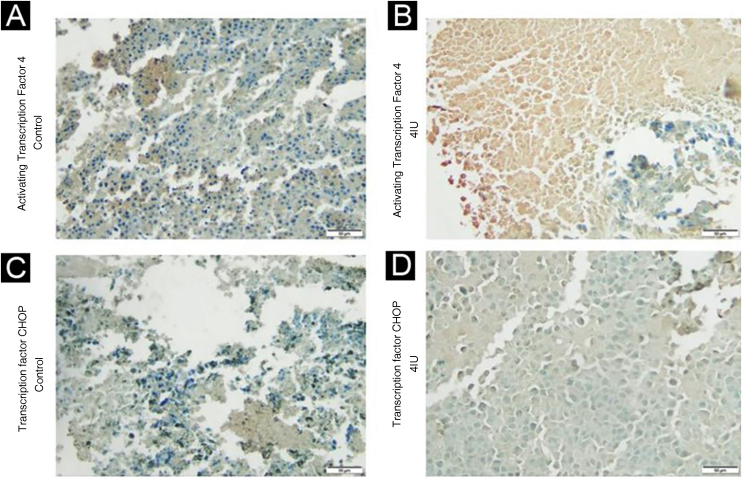
Immunohistochemical analysis of ATF4 and TFCHOP expression. Tissues obtained from tumor material from the control (A and C) and 4 IU/kg (B and D) groups were evaluated based on the immunohistochemical expression of these proteins. The photographs were obtained using an Axiolab 5 microscope (Zeiss) and analyzed using the ImageJ FIJI software program. The control and 4 IU/kg groups were chosen to represent dose extremes.

**Figure 3:**
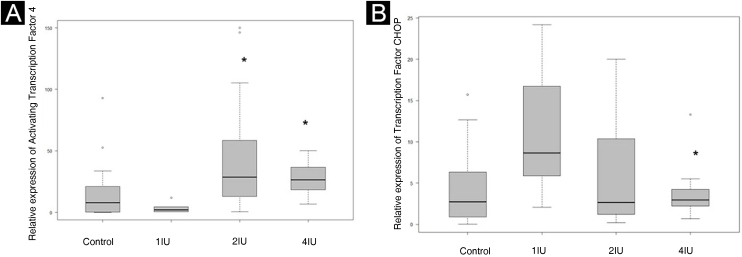
Relative expression of ATF4 and TFCHOP. Control slides were used to define the comparison parameters. (A) Quantification of the signal expressed by ATF4 in slides from different groups. (B) Quantification of the signal expressed by TFCHOP in slides from different groups. The dispersion of relative values was represented on the graph. Statistical evaluation was performed using the Kruskal-Wallis test, followed by Dunn’s test. * p value < 0.05 in relation to the control group.

**Table 1:** Immunohistochemical analysis of the expression of transcription factor CHOP and activating transcription factor 4 in tissues obtained from 1 IU/kg, 2 IU/kg and 4 IU/kg groups tumors. The data showed a lower number of nuclei in the groups treated with insulin, while a larger stained area was only observed in the evaluation of ATF4 and the 1IU group of TFCHOP. The p-value obtained by the Kruskal-Wallis test is represented in the last column, and values with a significant difference from the control group (p < 0.05) are marked with an asterisk.

	Control	1IU	2IU	4IU	p-value
**IHC Positive Area Average**					
ATF4	7.6%	2.0%	20.0%*	14.17%*	>0.05
**Number of nuclei per slide (average)**					
ATF4	1614	1345.7	721.0*	114.5*	>0.05
**IHC Positive Area Average**					
TFCHOP	1.7%	4.4%*	2.4%	1.5%	>0.05
**Number of nuclei per slide (average)**					
TFCHOP	2391	661.7*	785.9*	127.20*	>0.05

Although modern literature establishes a neoplastic role for insulin, so that the hormone use is related to tumor emergence/growth,^5^, ^6^ the present study is supported by data from the beginning of the 20^th^ century,^1^ in which it was demonstrated that insulin showed antineoplastic activity.

The insulin-mediated protein expression of ATF4, rather than hypoglycemia, seems to be related to antineoplastic action. ATF4 plays a dual role within the cell in situations of endoplasmic reticulum stress (ERS),^7^ promoting cell survival or initiating apoptosis, mainly through the PERK/eIF2a/ATF4/TFCHOP pathway. The literature stipulates that insulin is linked to ATF4 expression in healthy^8^ and tumor cells^9^ and these data are corroborated by the findings of the present article. The expression of TFCHOP only in the 4IU group may be related to the amount of tumor material in the slides of the 2IU group, observed by the stained percentage, as well as by other pathways for inducing apoptosis in cells, such as the TRAIL protein.^10^

The limitations of the present study include the fact that it did not assess whether the tumors would become refractory to insulin use or whether there would be recurrences. Animal weight, the possible increase in body mass in the groups treated with insulin, animals survival time after treatment, or the presence of other cell markers that could be involved in the processes, such as the TRAIL protein, were not evaluated. Another limitation is the evaluation of protein expression using immunohistochemical slides.

## Financial support

None declared.

## Authors’ contributions

Daniel do Prado: Design of the study; developed the theory behind the experiments; planned the study; drafting and editing of the manuscript; performed the statistical analysis and analyzed the data.

Marianna Boia: Performed the experiments; contributed to sample preparation and data interpretation.

Hanna Camara da Justa: Participated in the experiments.

Andrea Senff Ribeiro: Contributed to the interpretation of the results and supervised the project.

Sergio Lunardon Padilha: Contributed to the interpretation of the results and supervised the project.

## Conflicts of interest

None declared.
