# Supplementary material for: Insulin inhibits melanoma tumor growth through the expression of activating transcription factor 4, without detectable expression of transcription factor CHOP: an in vivo model
Source: An Bras Dermatol. 2024 Apr 23;99(4):587–91. doi: 10.1016/j.abd.2023.07.012 (PMC11221150; doi:10.1016/j.abd.2023.07.012)
Supplement: Supplementary file 1 [file mmc1.docx]

**ABD-D-23-00110**

**Supplementary Material**

**Supplementary Material 1** Animal treatment diagram.


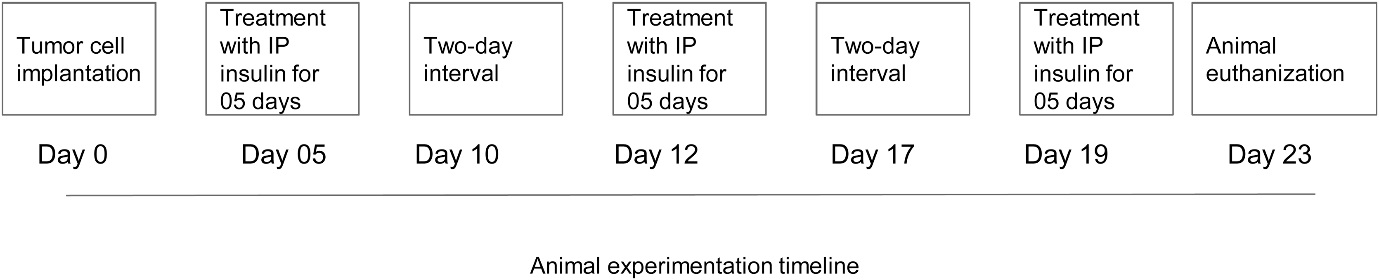


**Supplementary  Material 2** Detailed description of the method used for preparing immunohistochemistry slides.

| The immunohistochemical analysis was performed using slides of tumor sections from B16-F10 tumors obtained from *in vivo* experiments. The samples were fixed in 4% paraformaldehyde, in PBS, pH of 7.4, for 24 hours. Subsequently, the samples were dehydrated in increasing concentrations of ethanol, cleared in xylene and embedded in paraffin. Three-mcm sections of the samples were deposited on positively charged slides. Subsequently, the sections were deparaffinized in xylene, rehydrated in decreasing concentrations of ethanol; the rehydrated histological sections were incubated with 3% H_2_O_2_ in methanol to block endogenous peroxidase. Antigen retrieval was carried out in 10 mM sodium citrate buffer, with a pH of 6.0, at 100^o^C, for 20 minutes. Possible non-specific bindings, as well as free aldehyde radicals were blocked by incubation for five minutes with PBS containing 1% BSA and by PBS containing 0.1 M glycine, respectively. Subsequently, the sections were incubated for 12 hours at a controlled temperature of 2° to 8°C with the primary antibodies, anti-GADD153 for rats (Santa Cruz Biotechnology, cat. Sc 7351) and anti- Activating Transcription Factor 4 for rats, (Santa Cruz Biotechnology, cat. Sc 390063). After incubation with the primary antibody, the slides were washed in three PBS baths for five minutes each bath, and after that, incubated with the MACH1 polymer (Biocare Medical, MACH1TM) for 30 minutes. Subsequently, the binding of primary antibodies was revealed with DAB (“3,3’-diaminobenzidine tetrahydrochloride”) (DAB substrate Kit, BD Pharmigen), and subsequently counterstaining with hematoxylin. Next, the sections were dehydrated in an increasing battery of ethanol and then xylene, after which permanent slides were mounted with Entellan (Merck®). A total of 267 slides were obtained, 166 for GADD153 analysis and 201 for Activating Transcription Factor 4. Of these, 55 had tumor material on the slides: 16 from the control group, ten from the 1 IU/kg group, 19 from the 2 IU/kg group and ten from the 4 IU/kg group, while the analysis of Activating Transcription Factor 4 included 60 slides: 15 from the control group, five from the 1 IU/kg group, 30 from the 2 IU/kg group and ten from the 4 IU/kg group. Immunohistochemical positivity quantification was performed using the “ImageJ Analysis Software” program, according to the Crowe and Yue protocol. (4). Furthermore, the immunohistochemical positive area in the tumor and the number of nuclei per evaluated slide field were analyzed. |
| --- |
